# Supplementary material for: A Critical View of the Application of the APEX Software (Aqueous Photochemistry of Environmentally-Occurring Xenobiotics) to Predict Photoreaction Kinetics in Surface Freshwaters
Source: Molecules. 2019 Dec 18;25(1):9. doi: 10.3390/molecules25010009 (PMC7017383; doi:10.3390/molecules25010009)
Supplement: Supplementary file 1 [file molecules-25-00009-s001.zip › 3.2.4_gcc-4.4.0.zip!/3.2.4_gcc-4.4.0/3.2.4_gcc-4.4.0/doc/octave/html/interpreter/Return-Types-of-Operators-and-Functions.html]

Return Types of Operators and Functions - Untitled


Next: Mathematical Considerations,
Previous: Sparse Functions,
Up: Operators and Functions


---

##### 21.1.4.2 The Return Types of Operators and Functions

The two basic reasons to use sparse matrices are to reduce the memory
usage and to not have to do calculations on zero elements. The two are
closely related in that the computation time on a sparse matrix operator
or function is roughly linear with the number of non-zero elements.

Therefore, there is a certain density of non-zero elements of a matrix
where it no longer makes sense to store it as a sparse matrix, but rather
as a full matrix. For this reason operators and functions that have a
high probability of returning a full matrix will always return one. For
example adding a scalar constant to a sparse matrix will almost always
make it a full matrix, and so the example

```
     speye(3) + 0
     =>   1  0  0
       0  1  0
       0  0  1
```

returns a full matrix as can be seen.

Additionally, if `sparse_auto_mutate` is true, all sparse functions
test the amount of memory occupied by the sparse matrix to see if the
amount of storage used is larger than the amount used by the full
equivalent. Therefore `speye (2) * 1` will return a full matrix as
the memory used is smaller for the full version than the sparse version.

As all of the mixed operators and functions between full and sparse
matrices exist, in general this does not cause any problems. However,
one area where it does cause a problem is where a sparse matrix is
promoted to a full matrix, where subsequent operations would resparsify
the matrix. Such cases are rare, but can be artificially created, for
example `(fliplr(speye(3)) + speye(3)) - speye(3)` gives a full
matrix when it should give a sparse one. In general, where such cases
occur, they impose only a small memory penalty.

There is however one known case where this behavior of Octave's
sparse matrices will cause a problem. That is in the handling of the
diag function. Whether diag returns a sparse or full matrix
depending on the type of its input arguments. So

```
      a = diag (sparse([1,2,3]), -1);
```

should return a sparse matrix. To ensure this actually happens, the
sparse function, and other functions based on it like speye,
always returns a sparse matrix, even if the memory used will be larger
than its full representation.

— Built-in Function: val = **sparse\_auto\_mutate** ()  
— Built-in Function: old\_val = **sparse\_auto\_mutate** (new\_val)  
> Query or set the internal variable that controls whether Octave will
> automatically mutate sparse matrices to real matrices to save memory.
> For example,
>
> ```
>           s = speye(3);
>           sparse_auto_mutate (false)
>           s (:, 1) = 1;
>           typeinfo (s)
>           => sparse matrix
>           sparse_auto_mutate (true)
>           s (1, :) = 1;
>           typeinfo (s)
>           => matrix
> ```

Note that the `sparse_auto_mutate` option is incompatible with
matlab, and so it is off by default.
